# Rapamycin-Preactivated Autophagy Enhances Survival and Differentiation of Mesenchymal Stem Cells After Transplantation into Infarcted Myocardium

**DOI:** 10.1007/s12015-020-09952-1

**Published:** 2020-01-11

**Authors:** Zhi-hua Li, Yong-li Wang, Hai-jie Wang, Jin-hong Wu, Yu-zhen Tan

**Affiliations:** Department of Anatomy, Histology and Embryology, Shanghai Medical School of Fudan University, 138 Yixueyuan Road, Shanghai, 200032 People’s Republic of China

**Keywords:** Autophagy, Rapamycin, Mesenchymal stem cells, Stem cell transplantation, Myocardial infarction

## Abstract

Stem cell transplantation has been limited by poor survival of the engrafted cells in hostile microenvironment of the infarcted myocardium. This study investigated cytoprotective effect of rapamycin-preactivated autophagy on survival of the transplanted mesemchymal stem cells (MSCs). MSCs isolated from rat bone marrow were treated with 50 nmol/L rapamycin for 2 h, and then the cytoprotective effect of rapamycin was examined. After intramyocardial transplantation in rat ischemia/reperfusion models, the survival and differentiation of the rapamycin-pretreated calls were accessed. After treatment with rapamycin, autophagic activities and lysososme production of the cells were increased significantly. In the condition of short-term or long-term hypoxia and serum deprivation, the apoptotic cells in rapamycin-pretreated cells were less, and secretion of HGF, IGF-1, SCF, SDF-1 and VEGF was increased. After transplantation of rapamycin-pretreated cells, repair of the infarcted myocardium and restoration of cardial function were enhanced dramatically. Expression of *HGF*, *IGF-1*, *SCF*, *SDF-1*, *VEGF*, *HIF-1α* and *IL-10* in the myocardium was upregulated, while expression of *IL-1β* and *TNF-α* was downregulated*.* Tracing of GFP and *Sry* gene showed that the survival of rapamycin-pretreated cells was increased. Cardiomyogenesis and angiogenesis in the infarcted myocardium were strengthened. Some rapamycin-pretreated cells differentiated into cardiomyocytes or endothelial cells. These results demonstrate that moderate preactivation of autophagy with rapamycin enhances the survival and differentiation of the transplanted MSCs. Rapamycin-primed MSCs can promote repair of the infarcted myocardium and improvement of cardiac function effectively.

**Electronic supplementary material:**

The online version of this article (10.1007/s12015-020-09952-1) contains supplementary material, which is available to authorized users.

## Introduction

Myocardial infarction (MI) is an event of myocardial necrosis caused by an unstable ischemic syndrome. Necrosis of the myocardium triggers local inflammation, adverse remodelling and fibrosis of the ventricular wall. Finally, the patients die of heart failure or arhythmia. After the onset of myocardial ischemia, histological cell death takes a finite period of time to develop as little as 20 min or less in some animal models. The entire process leading to a healed infarction after reperfusion usually takes at least 5–6 weeks [1]. MI mortality has declined following modern conventional therapies including pharmacotherapy, percutaneous coronary intervention and coronary artery bypass grafting [2]. However, these therapies are almost ineffective for myocardial regeneration. Although mobilization of endogenous stem cells and division of the survived cardiomyocytes are involved in heart regeneration, this process is very slow in cardiomyocyte replacement [3]. The heart with MI may lose one billion cardiomyocytes, approximately 25% of its mass [4]. Renewal of cardiomyocytes is up to 1% per year at the age of 25 to 0.45% at the age of 75 [5]. Therefore, stem cell-based therapies have become promising approaches in myocardial regeneration in recent years [6].

Mesemchymal stem cells (MSCs) have been considered a very effective cell population for cardiac transplantation. MSCs may be isolated easily from bone marrow or adipose tissue, and have a high self-renewal capacity, and can differentiate into cardiomyocytes, endothelial cells and vascular smooth muscles. Moreover, MSCs possess immunomodulatory properties [7]. Nevertheless, only ~ 5% of MSCs survived after transplantion into the infarcted myocardium [8]. Intraarterially delivered MSCs decreased to 14% of the initial numbers at day 3 [9]. Intramyocardially injected MSCs did not present in the scar of the infarcted myocardium at 4 weeks [10]. Therefore, efficiency of stem cell-based therapy in experimental animal models as well as clinical trails has been less than optimal under massive death of the engrafted cells. Evidently, it is important to improve survival of the transplanted cells for repair of the infarcted myocardium. Approaches priming stem cells into prosurvival state include heat shock, hypoxia, cytokine stimulation, genetic modification and treatment with pharmacologics [7, 11].

Autophagy is an evolutionarily conserved process that maintaining cell survival by degradation of organelles and cytoplasmic proteins in response to various forms of stress such as ischemia, reperfusion and inflammation [12]. It may be divided into macroautophagy (hereafter referred to as autophagy), microautophagy and chaperone-mediated autophagy based on the pathways by which cargos are delivered into lysosomes [13]. Autophagy plays important roles in maintaining metabolism and function [14], regulating growth and differentiation [15], and preventing ROS (reactive-oxygen species)-induced senescence of stem cells [16]. Our previous study suggested that hypoxia stimulates autophagy, promoting the survival of endothelial progenitor cells (EPCs) via inhibiting apoptosis [17]. Hypoxic precondition-induced autophagy enhanced the survival of the engrafted EPCs in ischemic limb [18]. However, some cells become apoptotic during hypoxic precondition. Interestingly, it is well known that rapamycin is a specific and potent activator of autophagy [19]. Effectiveness of precondition with rapamycin on enhancing survival and differentiation of the transplanted stem cells remains to be investigated.

This investigation was designed to examine cytoprotective effects of autophagy activated with rapamycin in vitro experiments and evaluate survival and differentiation of rapamycin-primed MSCs in rat ischemia/reperfusion (I/R) models. Here we demonstrate that rapamycin can activate autophagy of MSCs effectively and protects the cells from apoptosis in the condition of hypoxia and serum deprivation. Paracrine of the cells is increased after treatment with rapamycin. Pretreatment with rapamycin promotes survival and differentiation of the engrafted cells. Transplantation of rapamycin-pretreated cells enhances cardiomyogenesis, angiogenesis and improvement of cardiac function. Therefore, preactivation of autophagy with rapamycin is a feasible strategy for MSC transplantation.

## Methods

### Isolation of MSCs

Sprague-Dawley (SD) rats were obtained from Department of Laboratory Animal Science of Fudan University. Isolation of MSCs from bone marrow of male SD rats was performed as described previously [20]. In brief, the rats (3–4 weeks old) were underwent chamber induction with 4% isoflurane mixed with 1 L/min air, and then sacrificed by cervical dislocation. Bone marrow in the femurs and tibias was flushed out with PBS containing heparin (100 U/ml). The cells of bone marrow were incubated with Dulbecco’s modified Eagle’s medium (DMEM; Invitrogen, Carlsbad, CA, USA) supplemented with 15% fetal bovine serum (FBS; Invitrogen). At 24 h after incubation, the non-adherent cells were removed, and then the adherent cells continued to be incubated with complete medium. The isolated MSCs were identified with specific expression of CD29, CD44, CD90, CD105 and Scal-1 after analysis using BD FACSVerse™ flow-cytometer (BD Biosciences, San Jose, CA, USA). The cells of the third to fifth passages were used in following experiments.

### Western Blotting

Autophagic activity of the cells was evaluated by LC3 (microtubule-associated protein 1 light chain 3) detection with immunoblotting after treatment with 50 nmol/L rapamycin (Sigma, CA, USA) for 2 h. Total cell proteins were extracted with RIPA buffer (Beyotime, Suzhou, China). Protein concentration was determined by BCA assay (Beyotime). Protein exacts were separated using 12% sodium dodecyl sulfate polyacrylamide gel electrophoresis (SDS-PAGE; Beyotime) and transferred onto ployvinylidene fluoride membrane (EMD Millipore, Billerica, MA, USA). Then, the membrane was blocked with 5% non-fat milk in Tris-buffered saline containing 0.1% Tween 20 (TBST) for 30 min at room temperature. After incubation with rabbit anti-rat LC3 antibody (1:500, Novus, Littleton, CO, USA) and mouse anti-rat β-actin antibody (1:3000, EMD Millipore) at 4 °C overnight respectively, the membrane was incubated with HRP-conjugated goat anti-rabbit antibody (1:5000) and HRP-conjugated goat anti-mouse antibody (1:1000; Beyotime) at room temperature for 2 h. After washing in TBST for three times, the blots was detected by BeyoECL (Beyotime) and imaged in ChemiDoc XRS+ system (Bio-Rad Laboratories, Hercules, CA, USA). Relative expression of LC3-II was shown as a ratio of LC3-II/β-actin.

### LC3 Immunostaining

LC3 is expressed mainly on autophagosome and is used as a specific marker for autophagic structures [21]. After treatment with rapamycin for 2 h, the cells were fixed in 4% paraformaldehyde, and permeabilized with 0.5% Triton X-100. Then, the cells were incubated with rabbit anti-LC3 antibody (1:100; Abcam, Cambridge, MA, USA) overnight at 4 °C and goat anti-rabbit IgG conjugated with FITC (1:200; BD Biosciences, San Jose, CA, USA) at room temperature for 1 h. The nuclei were stained with 4, 6-diamidino-2-Phenylin-phenylindole (DAPI, 1:1000; Sigma-Aldrich, St Louis, MO, USA) for 10 min. LC3-positive puncta were viewed with a fluorescence microscope, and counted in five fields randomly. The experiment was repeated for three times.

### Transmission Electron Microscopy

Rapamycin-treated cells were fixed overnight in 3% glutaraldehyde and post-fixed in 1% osmic acid for 2 h. Subsequently, a series of dehydration was performed. The cells were embedded in epoxy resin. Ultrathin sections were made by an ultra-microtome. After being stained with lead citrate and uranyl acetate, the autophagic structures in the cells were viewed using a transmission electron microscope (Philips Electronic Instruments, Mahwah, NJ, USA). The ratio of the cross-section area of the autophagic structures to that of the cytoplasm was calculated. The autophagic structures were examined in 200 cells for each group.

### Labeling of Lysosomes

After treatment with rapamycin, the cells were incubated with 50 nmol/L LysoTracker Red (Molecular Probes, Carlsbad, CA, USA) for 30 min. Following DAPI (1: 200) was added into the medium, the cells continued to be incubated for 30 min. Lysosomes were viewed with a confocal laser scanning microscope (Leica Microsystems, Wetzlar, Germany). The fluorescent density of the lysosomes was analyzed in five sequential 400× fields per culture dish. The experiment was repeated for three times.

### Ethidium Bromide/Acridine Orange (EB/AO) Staining

To mimic short-term or long-term ischemia, the cells were incubated in the conditions of hypoxia and serum free for 2 h and 12 h respectively. For conditioning with hypoxia, the culture dishes were put into a hypoxic chamber (Billups-Rothenberg, San Diego, CA, USA) flushed with a gas mixture of 1% O_2_, 5% CO_2_ and 94% N_2_. The cells were divided into control, rapamycin and 3-methyladenine (3-MA) groups. 3-MA, an inhibitor of class III PI3K, inhibits formation of autophagosome [22]. The cells were pretreatment with 50 nmol/L rapamycin or 5 mmol/L 3-MA (Sigma) for 2 h. Apoptosis and necrosis of the cells were examined with EB/AO staining. The percentage of apoptotic and necrotic cells were calculated respectively by the following formula: the percent (%) = number of early apoptotic, late apoptotic and necrotic cells/number of all counted cells. The number of survived cells was calculated by ImageJ. Five random fields (10×) were selected in each dish. Two dishes were conducted simultaneously. The experiments were repeated for three times.

### MTT Assay

Viability of the cells was assessed with MTT [3-(4,5-Dimethylthiazol-2-Yl)-2,5-Diphenyl-2Htetrazolium Bromide] assay. The cells were seeded in a 96-well plate, Three parallel wells were for each group. The cells were pretreatment with rapamycin or 3-MA for 2 h and then incubated in the conditions of hypoxia (1% O_2_) and serum free for 24 h. Subsequently, 10 μL MTT (5 mg/mL; Dojindo, Kumamoto, Japan) was added to the wells, and the cells continued to be incubated for 4 h. after sucking up the supernatant, 100 μL DMSO was added to fully dissolve the generated formazan crystal. Absorbance values at 570 nm were measured using an Infinite 200 PRO Microplate Reader (Tecan, Mannedort, Switzerland)

### Enzyme-Linked Immunosorbent Assay (ELISA)

The cells pretreated with rapamycin were incubated in 1% O_2_ and serum-free DMEM for three, five and seven days respectively. Hepatocyte growth factor (HGF), insulin-like growth factor 1 (IGF-1), stem cell factor (SCF), stromal cell-derived factor 1 (SDF-1) and vascular endothelial growth factor (VEGF) in the supernatant were detected with ELISA Kit (Guchen Biotechnology, Shanghai, China), and absorbance values were measured with a microplate reader (Tecan Infinite 200; Tecan).

### Establishment of Ischemia/Reperfusion Model and Cell Transplantation

The protocol establishing rat models of cardiac ischemia/reperfusion (I/R) complied with the institution’s guidelines as above. Female SD rats (200–250 g) were anesthetized by intraperitoneal injection of ketamine (80 mg/kg) and xylazine (5 mg/kg). After endotracheal intubation and ventilation using a rodent ventilator, thoracotomy was performed at the left third intercostal space. The left anterior descending coronary artery (LADCA) was double ligated for 30 min. The ligation was deemed successful when the free wall of the left ventricle (LV) became pale and contractility of the heart was reduced. Then, the knots were released to initiate reperfusion, and the chest was closed [23]. At 1 week after reperfusion, thoracotomy was performed at the fourth intercostal space, and 2 × 10^6^ cells in 80 μL PBS were injected into the border of the infarcted myocardium at four spots with an insulin syringe. Injection of same volume PBS in the infarcted rats and sham-operated rats was used as control. Pale blebs in the area of injection were clearly visible for proper injection.

### Echocardiography

Echocardiographic studies were performed before MI, at 1 week after MI and at 4 weeks after cell transplantation. The rats were divided into sham surgery (*n* = 6), control, MSC and rapamycin (*n* = 10) groups and anaesthetized in an induction chamber with 4% isoflurane (Sinopharm Chemical Reagent Company, Shanghai, China) mixed with 1 L/min air. The anaesthesia during the ultrasound measurement was maintained at 1.5% isoflurane with 1 L/min air. A commercial echocardiographic machine (VisualSonics, Toronto, Canada) with a 15 MHz linear transducer was used for evaluation of dimension and systolic function of LV. After adequate two-dimensional images were obtained, the M-mode cursor was positioned to the parasternal long axis view at the level of the papillary muscles. LV end-diastolic diameter (LVEDD) and LV end-systolic diameter (LVESD) were measured from at least 3 consecutive cardiac cycles. For examining systolic function, LV end-diastolic volume (LVEDV), LV end-systolic volume (LVESV), ejection fraction (EF = LVEDV - LVESV/LVEDV × 100%) and fractional shortening (FS = LVEDD - LVESD/LVEDD × 100%) were measured. Two echocardiographers blinded to the experimental treatment acquired the images.

### Quantitative Reverse Transcription-Polymerase Chain Reaction (qRT-PCR)

To evaluate effect of autophagy pre-activated with rapamycin on paracrine of cardiac tissue after cell transplantation, qRT-PCR was performed on a StepOnePlus Real-Time PCR System (Applied Biosystems) with Fast SYBR Green Master Mix (Takara, Otsu, Japan). The rats were divided into control, MSC and rapamycin groups (*n* = 6). The left ventricular wall at peri-infarct area was removed at 7 days after cell transplantation. Expression of *HGF*, *IGF-1*, *SCF*, *SDF-1*, *VEGF*, *HIF-1α* (*hypoxia-inducible factor 1-α*), *IL-1β* (*interlokin-1β*), *IL-10* and *TNF-α* (*tumor-necrosis factor-α*) was examined with qRT-PCR. The detected genes and their primer sequences were described in Table S1 (Additional file 1).

### Masson’s Trichrome Staining

The hearts were harvested at three days or four weeks after cell transplantation. According the approach previously described [24], the heart was arrested in diastole by an intravenous injection of 1 mL KCl solution (1 mol/L) through the jugular vein. Then, the thorax was opened, and the heart was fixed by perfusion with 4% paraformaldehyde. The heart was cut into upper, middle and lower parts along cross-axis section. The cryostat cryosections of 5 μm thickness were prepared. Discontinuous sections of the hearts at 4 weeks after cell transplantation were stained with Masson’s trichrome. The collagen-rich scar tissue in the infarcted LV wall was stained blue, while viable myocardial tissue was stained red. Scar size was calculated as percentage of circumference of the maximum blue region in circumference of whole LV wall. The thickness of LV wall was measured at the minimum thickness region of the infarcted LV wall.

### Identification of the Engrafted Cells

The rats were divided into MSC, rapamycin and 3-MA groups (*n* = 8). In rapamycin and 3-MA groups, the cells were treated with rapamycin and 3-MA for 2 h before transplantation respectively. For tracing the transplanted cells, the cells were transfected with lentiviruses carrying GFP (green fluorescent protein) expression cassette. For measuring the DNA copy of Sry gene on the Y chromosome of the engrafted cells, whole LV anterior wall was collected at 3 days after transplantation, and digested with 1 mL lysis buffer containing 1% proteinase K for overnight. 100 μL of the digested solution was used to isolate total DNA to count MSC number in each milligram. Primer sequences for *Sry* gene were AGTGTTCAGCCCTACAGCCTGAGGAC (forward) and GTGTGTAGGTTGTTGTCCCATTGCAGC (reverse). The size of the PCR products was 411 bp. Reaction conditions were 94 °C, 74 °C and 72 °C, 1 min each, 40 cycles. The PCR products were analyzed on 1% agarose gel and visualized under ultraviolet light following EB staining. The cell number in each milligram will be calculated based on the cycle number of experimental DNA after RT-PCR [25].

### Immunostaining of the Myocardium

For assessing survival and distribution of the transplanted cells, GFP immunostaining was performed on the sections obtained from hearts at 3 days and 4 weeks after cell transplantation respectively. The sections were incubated with rabbit anti-rat GFP antibody (1:200; Santa Cruz, Dallas, TX, USA) at 4 °C overnight, followed by incubation with Alexa fluor 594-labelled goat anti-rabbit IgG or Alexa fluor 488-labelled goat anti-rabbit IgG (1:400; Jackson, West Grove, PA, USA) for 1 h at room temperature. The nuclei were counterstained with DAPI (1:1000). Survival of engrafted cells was determined by counting GFP^+^ cells from three independent sections of the upper, middle and lower parts of the infarct area. Five fields (20×) were randomly selected in each section. To assess differentiation of the transplanted cells towards cardiomyocytes and endothelial cells, co-expression of GFP and cTnT or CD31 was determined by double immunostaining. The sections were incubated with rabbit anti-rat GFP antibody and mouse anti-cTnT antibody (1:200; Santa Cruz) or mouse anti-CD31 antibody (1:200; Abcam, Cambridge, MA, USA). Then, the sections were incubated with Alexa fluor 488-labelled goat anti-rabbit IgG and Alexa fluor 594-labelled goat anti-mouse IgG (1:400; Jackson). Differentiation of the GFP^+^ cells into cardiomyocytes was determined by observing GFP^+^cTnT^+^ cells. Density of the microvessels in the infarct region was examined by counting CD31-positive structures from three independent sections of the middle part of the infarct area. Five fields (20×) were randomly selected in each section.

### Statistical Analysis

Results are presented as means ± standard error unless otherwise stated. Significance between two measurements was determined by Student’s t test, and in multiple comparisons was evaluated by the Bonferroni method. Values of *P* < 0.05 were considered significant.

## Results

### Phenotypic Characteristics of Bone Marrow-Derived MSCs

The flow cytometric results demonstrated that the isolated MSCs were negative for hematopoietic markers (CD34 and CD45) and expressed mesenchymal lineage markers (CD29, CD44, CD90, CD105 and Scal-1) specifically (Fig. S1).

### Activation of Autophagy after Treatment with Rapamycin

**Fig. 1:**
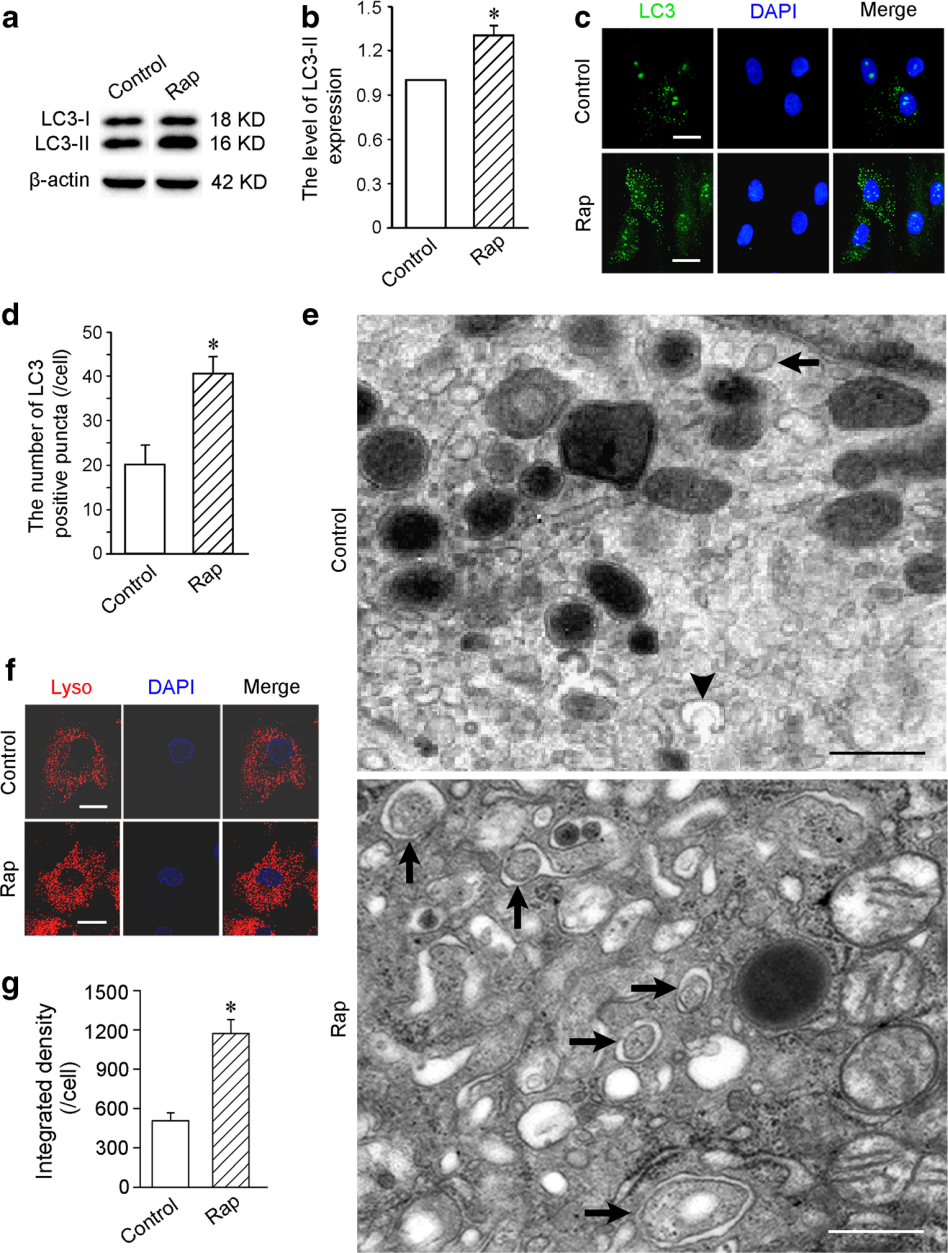
The changes of LC3 expression and autophagic structures. After treatment with 50 nmol/L rapamycin for 2 h, autophagic activities of the cells are activated significantly. **a** The levels of LC3-I and LC3-II expression. Western blot. **b** Statistic result of the level of LC3-II expression. **c** LC3-positive puncta. Scale bar = 20 μm. **d** Statistic result of LC3-positive puncta. **e** Representative autophagic ultrastructures. Arrowhead and arrows indicate autophagosome precursor and autophagosomes with double membranes respectively. Scale bar = 500 nm. **f** Lysosomes labeled with LysoTracker Red. Scale bar = 10 μm. **g** Statistic result of the fluorescent density of lysosomes. *n* = 3. *p < 0.01 versus control group. Rap, rapamycin

The result of Western blot showed that LC3-II expression in the cells treated with rapamycin for 2 h was increased significantly (Fig. 1a, b). Compared with control group, the number of LC3-positive puncta in rapamycin group was greater (Fig. 1c, d). After induction with rapamycin, the autophagic ultrastructures in the cells were increased. Representative electron micrographs of the autophagic ultrastructures were showed in Fig. 1e. The ratio of the cross-section area of the autophagic ultrastructures to that of the cytoplasm in rapamycin group (21.25 ± 3.62) was higher than control group (3.52 ± 0.55, *p <* 0.01). Moreover, the fluorescent density of lysosomes in rapamycin-treated cells was higher (Fig. 1f, g).

### Cytoprotective Effect of Rapamycin-Activated Autophagy

**Fig. 2:**
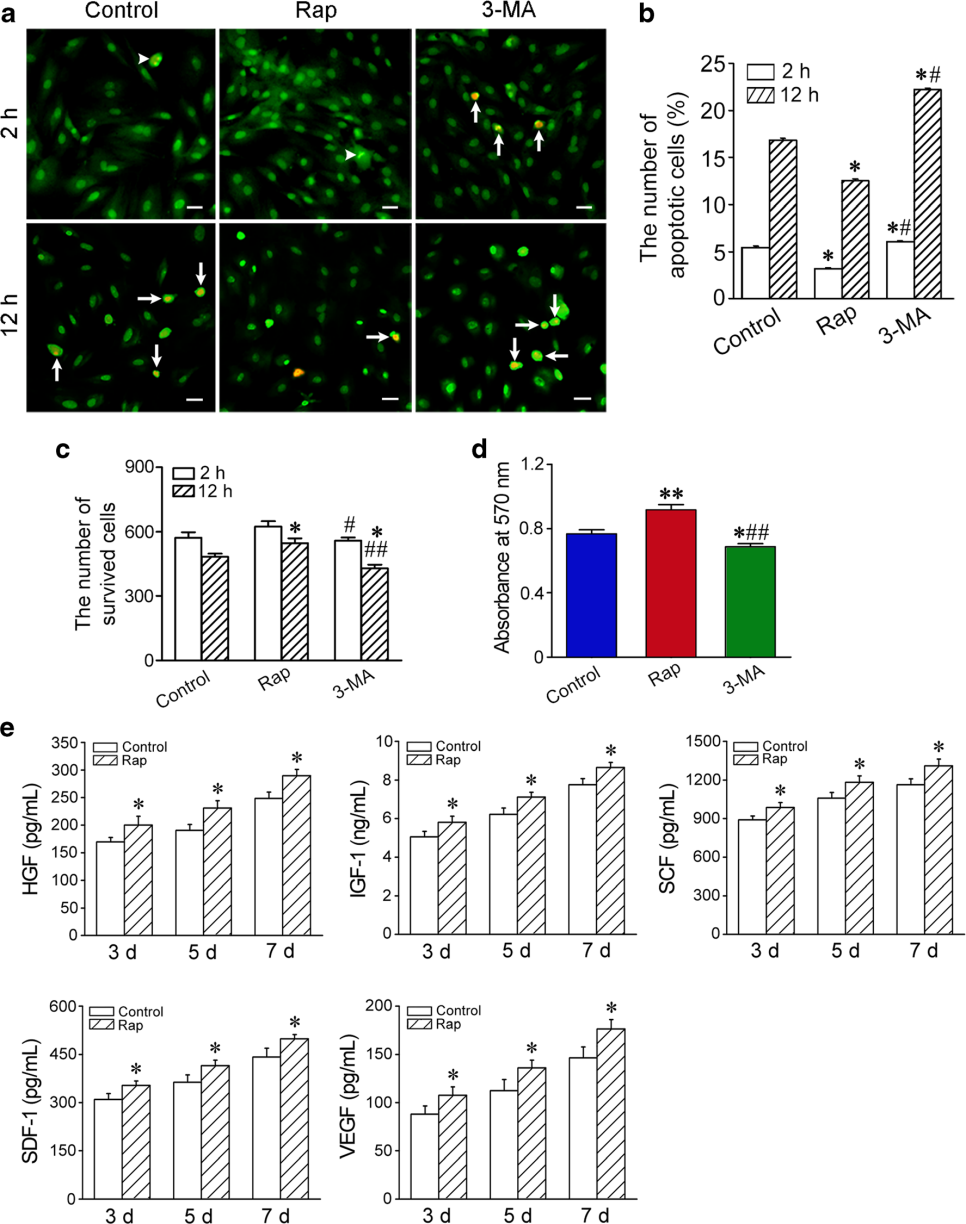
The survival and paracrine of the cells in the condition of hypoxia and serum deprivation. **a** The apoptotic cells. The cells were pretreated with rapamycin or 3-MA for 2 h and then incubated in the condition of hypoxia and serum deprivation for 2 h or 12 h respectively. Arrowheads and arrows indicate early and late apoptotic cells respectively. EB/AO staining. Scale bar = 20 μm. **b** Statistic result of the apoptotic cells. *n* = 3. *p < 0.01 versus control group; ^#^*p <* 0.01 versus rapamycin group. **c** Statistic result of the survival cells. **d** Viability of the cells. *n* = 3. *p < 0.05 and **p < 0.01 versus control group; ^#^*p <* 0.05 and ^##^*p <* 0.01 versus rapamycin group. **e** The concentrations of HGF, IGF-1, SCF, SDF-1 and VEGF released from the cells. *n* = 5. * *p* < 0.05 versus control group

At 2 h and 12 h after incubation in the conditions of hypoxia and serum deprivation, the apoptotic cells in rapamycin-pretreated cells were lesser than that in the cells of control group. However, the apoptotic cells in 3-MA-pretreated cells were significantly more than that in the cells of control and rapamycin groups (Fig. 2a, b). The number of the survived cells in rapamycin group was greater than that in control group at 12 h after hypoxia and serum deprivation. The number of the survived cells in 3-MA group was smaller than that in control and rapamycin groups at 12 h after hypoxia and serum deprivation. The survived cells in 3-MA group were less than that in rapamycin group at 2 h after hypoxia and serum deprivation (Fig. 2c). The result of MTT showed that viability of the cells in rapamycin group was higher than that in control group at 24 h after hypoxia and serum deprivation. Viability of the cells in 3-MA group was significantly lower than that in control and rapamycin groups (Fig. 2d).

### Paracrine of the Rapamycin-Treated Cells

In the condition of hypoxia and serum deprivation, the concentrations of HGF, IGF-1, SCF, SDF-1 and VEGF in the supernate of the cells increased gradually in control and rapamycin groups. Compared with control group, concentrations of the factors in rapamycin group were higher at three, five and seven days after incubation respectively (Fig. 2e).

### Expression of Paracrine Factor mRNAs in the Infarcted Myocardium

For evaluating the changes of mRNA expression of paracrine factors in myocardial tissue after cell transplantation, heart tissue samples were obtained 1 week after transplantation. The results of qRT-PCR detection showed that expression of *HGF*, *IGF-1*, *SCF*, *SDF-1*, *VEGF*, *HIF-1α* and *IL-10* in MSC and rapamycin groups was increased significantly compared with control group. Expression of the genes in rapamycin group was higher than that in MSC group. In MSC and rapamycin groups, expression of *IL-1β* and *TNF-α* was decreased*.* Difference in expression of *IL-1β* and *TNF-α* between these two groups was significant (Additional file 1: Fig. S2).

### Improvement of Cardiac Function after Cell Transplantation

**Fig. 3:**
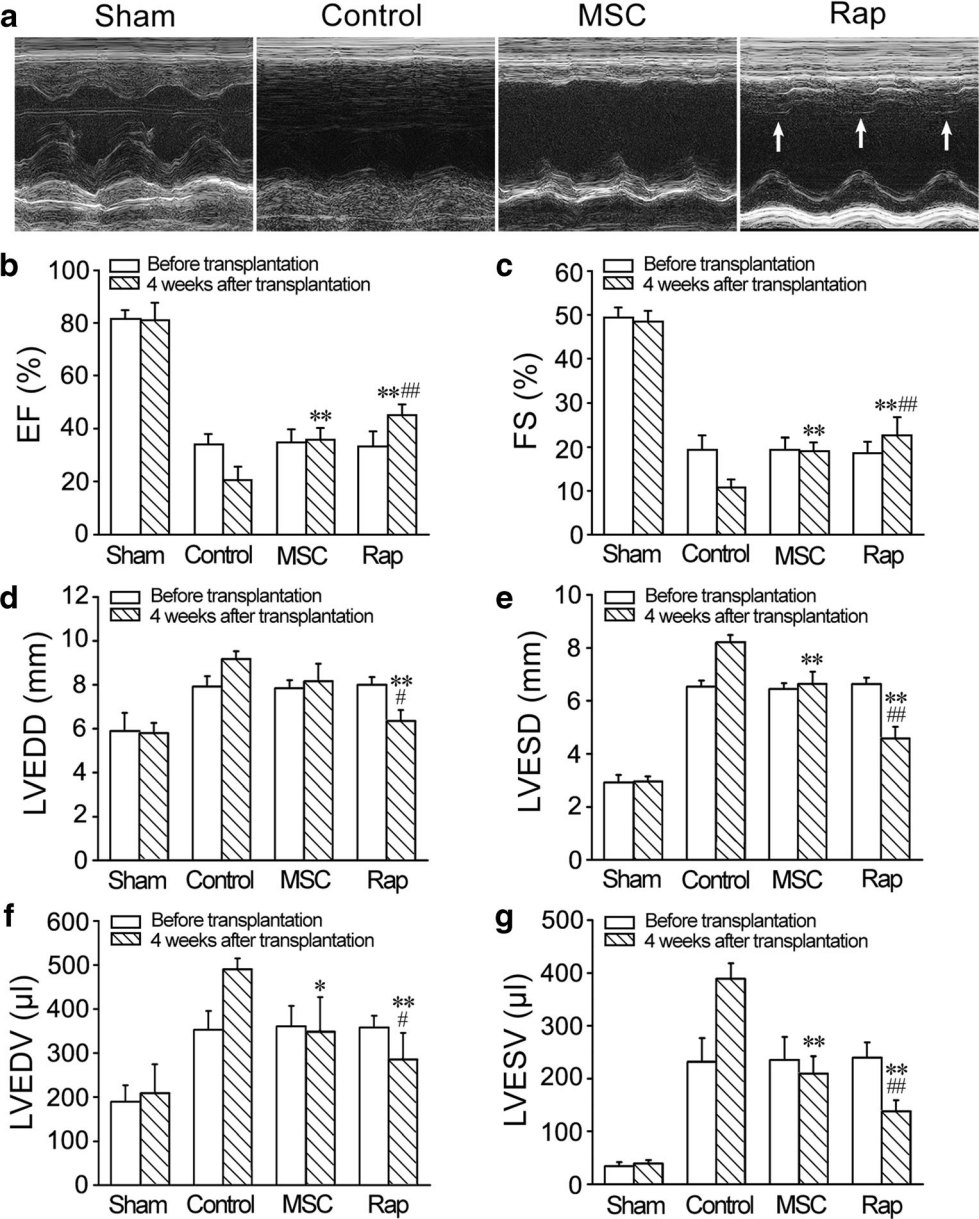
Improvement of cardiac function after cell transplantation. **a** Representative echocardiograms of the LV free walls. LV contraction in rapamycin group was significantly improved (arrows). **b–g** Statistic results of EF, FS, LVEDD, LVESD, LVEDV and LVESV. *n* = 6. *P < 0.015, **p < 0.01 versus control group; ^#^*P* < 0.015, ^##^*p* < 0.01 versus MSC group

Echocardiography revealed that cardiac function in all rats was severely compromised at 1 week after I/R. In control group, cardiac functional loss lasted for following 4 weeks. Echocardiography revealed that Function of the heart implemented cell transplantation was significantly improved at 4 weeks (Fig. 3a). EF and FS were significantly increased in MSC and rapamycin groups. Compared with MSC group, EF and FS in rapamycin group were greater (Fig. 3b, c). LVEDD, LVESD, LVEDV and LVESV were obviously decreased in rapamycin group compared with that in the control and MSC groups (Fig. 3d–g).

### Histological Changes of the LV Wall after Cell Transplantation

**Fig. 4:**
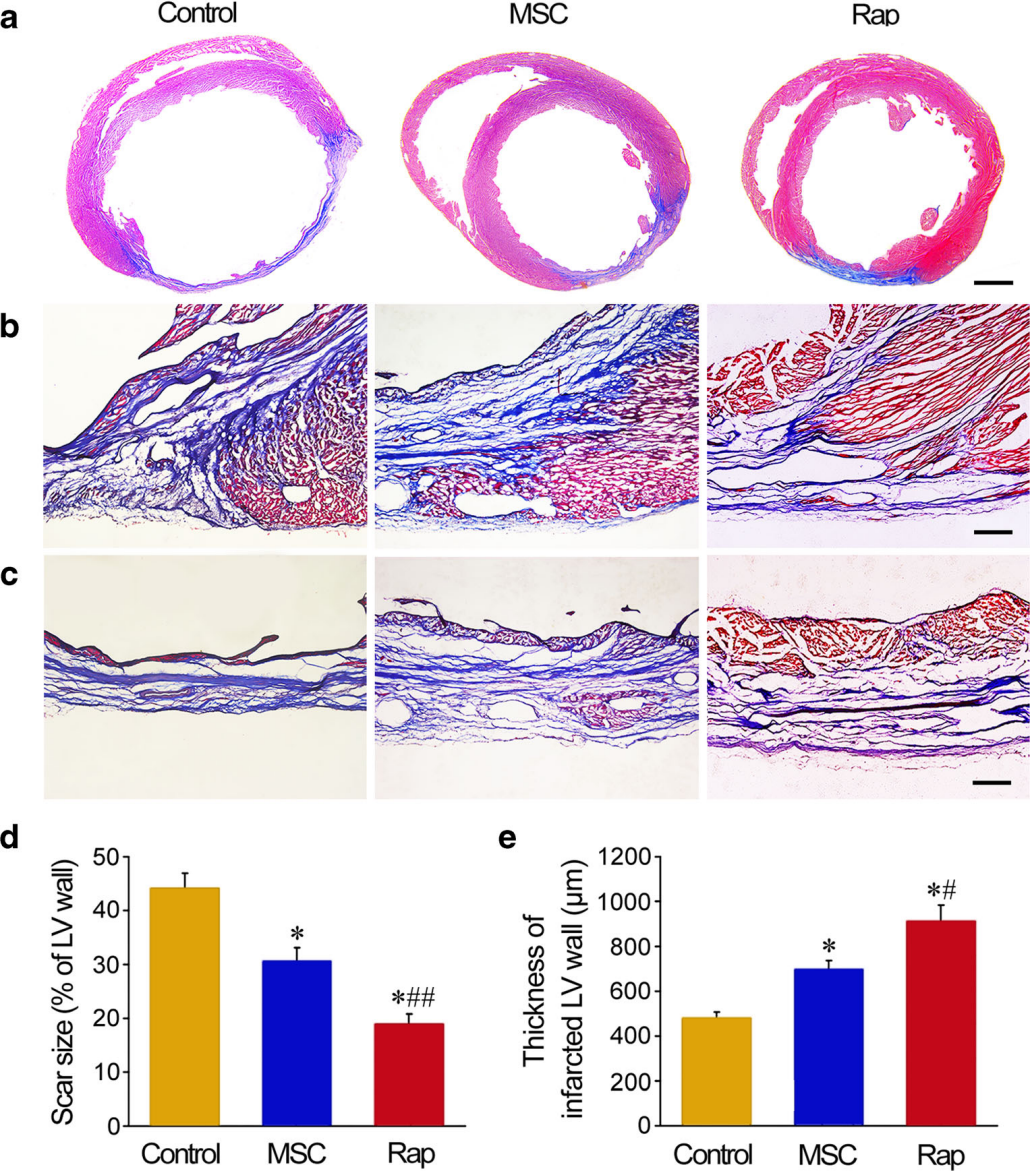
Structural changes in LV wall after cell transplantation. **a–c** The transverse sections of the ventricles at the widest part of the infarct region. At 4 weeks after cell transplantation, the sections were stained with Masson’s trichrome. The collagen-rich scar tissue was stained blue, while viable myocardial tissue was stained red. Scale bars = 2 mm (**a**), 100 μm (**b**, **c**). **d, e** Statistic results of scar size and thickness of LV wall. *n* = 6. *P < 0.01 versus control group; ^#^*P* < 0.05 and ^##^*P* < 0.01 versus MSC group

In rapamycin group, there was more myocardial tissue at the infarct region (Fig. 4a–c). Quantitative analysis demonstrated that the size of scar was smaller, and the thickness of LV wall was greater in MSC and rapamycin groups than that in control group. Compared with MSC group, the size of scar was decreased, and the thickness of LV wall was increased significantly in rapamycin group (Fig. 4d, e).

### The Survived and Differentiated Cells in the Engrafted Cells

**Fig. 5:**
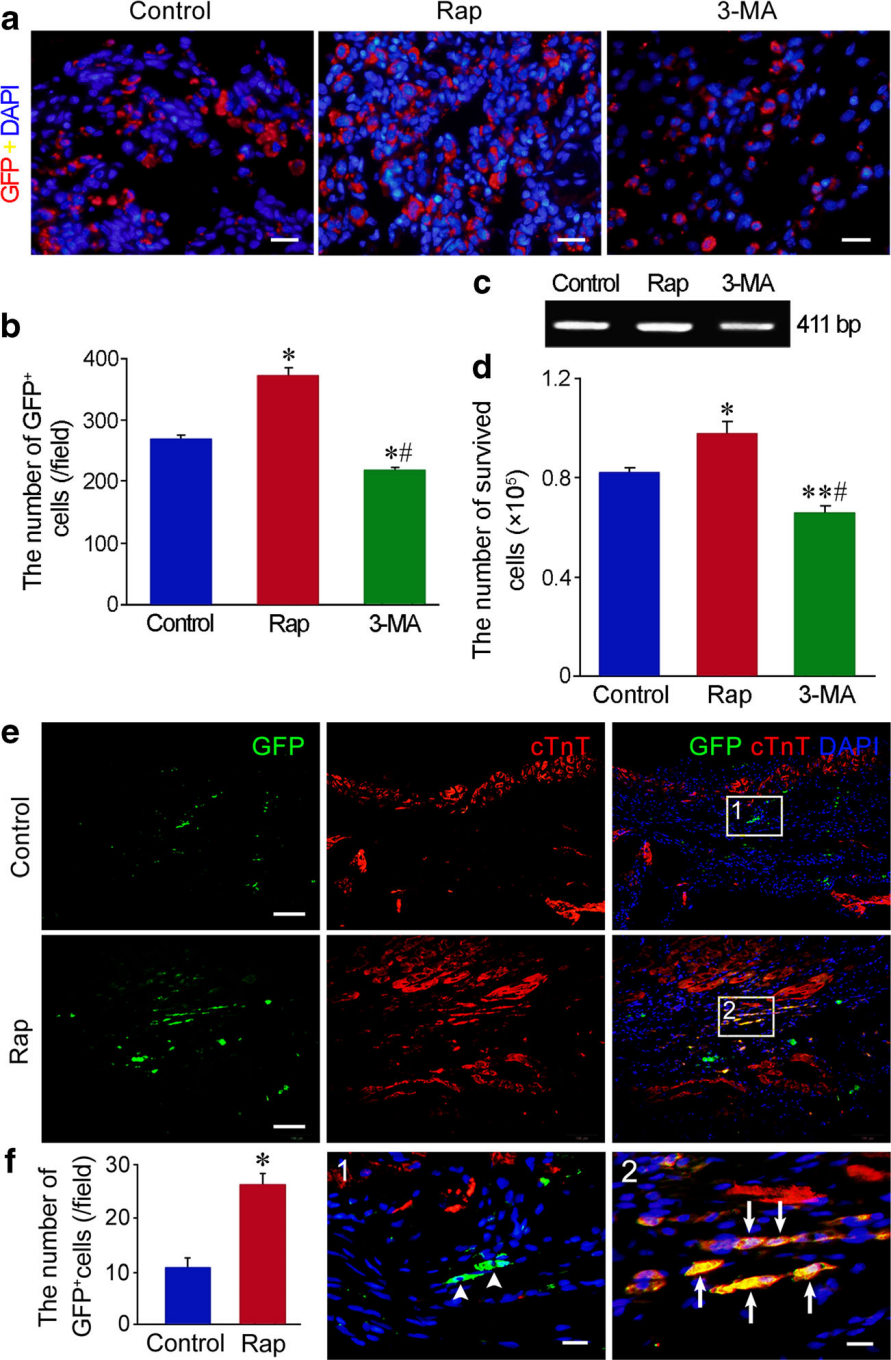
The survival and differentiation of the cells after transplantation. **a** The survived cells (GFP^+^ cells) in the engrafted cells at the peri-infarct region at 3 days after transplantation. Scale bar = 20 μm. **b** Statistic result of the number of GFP^+^ cells at the peri-infarct region. n = 6. *p < 0.01 versus control group; ^#^*p <* 0.01 versus rapamycin group. **c** RT-PCR analysis of *Sry* gene in the anterior wall of LV at 3 days after cell transplantation. **d** Statistic result of the cells expressing *Sry* gene. *n* = 3. *p < 0.05 and **p < 0.01 versus control group; ^#^*p <* 0.01 versus rapamycin group. **e** The survived cells in the engrafted cells and their differentiation towards cardiomyocytes at the infarct region at 4 weeks after transplantation. Some GFP^+^ cells express cTnT in rapamycin group. The panels of the third line are magnification of the boxes in the upper panels. Scale bar = 100 μm (the panels of the first and second lines) and 20 μm (the panels of the third line). **f** Statistic result of the number of GFP^+^ cells at the infarct region. *n* = 6. *p < 0.01 versus control group

The survival of the engrafted cells was evaluated by GFP tracing and *Sry* gene detection. At 3 days after transplantation, GFP^+^ cells at the peri-infarct region in rapamycin group were more than that in control group. Compared with control and rapamycin groups, the number of GFP^+^ cells in 3-MA group was reduced (Fig. 5a, b). RT-PCR result showed that *Sry* gene at the anterior wall of LV in rapamycin group was more than that in control group at 3 days after transplantation. After autophagy was inhibited with 3-MA, *Sry* gene was decreased significantly (Fig. 5c, d). At 4 weeks after transplantation, GFP^+^ cells at the infarct region in rapamycin group were more than that in MSC group. GFP and cTnT double immunostaining showed that some GFP^+^ cells expressed cTnT in rapamycin group. GFP^+^cTnT^+^ cells were not observed in control group (Fig. 5e, f; Fig. S3).

### Angiogenesis after Cell Transplantation

**Fig. 6:**
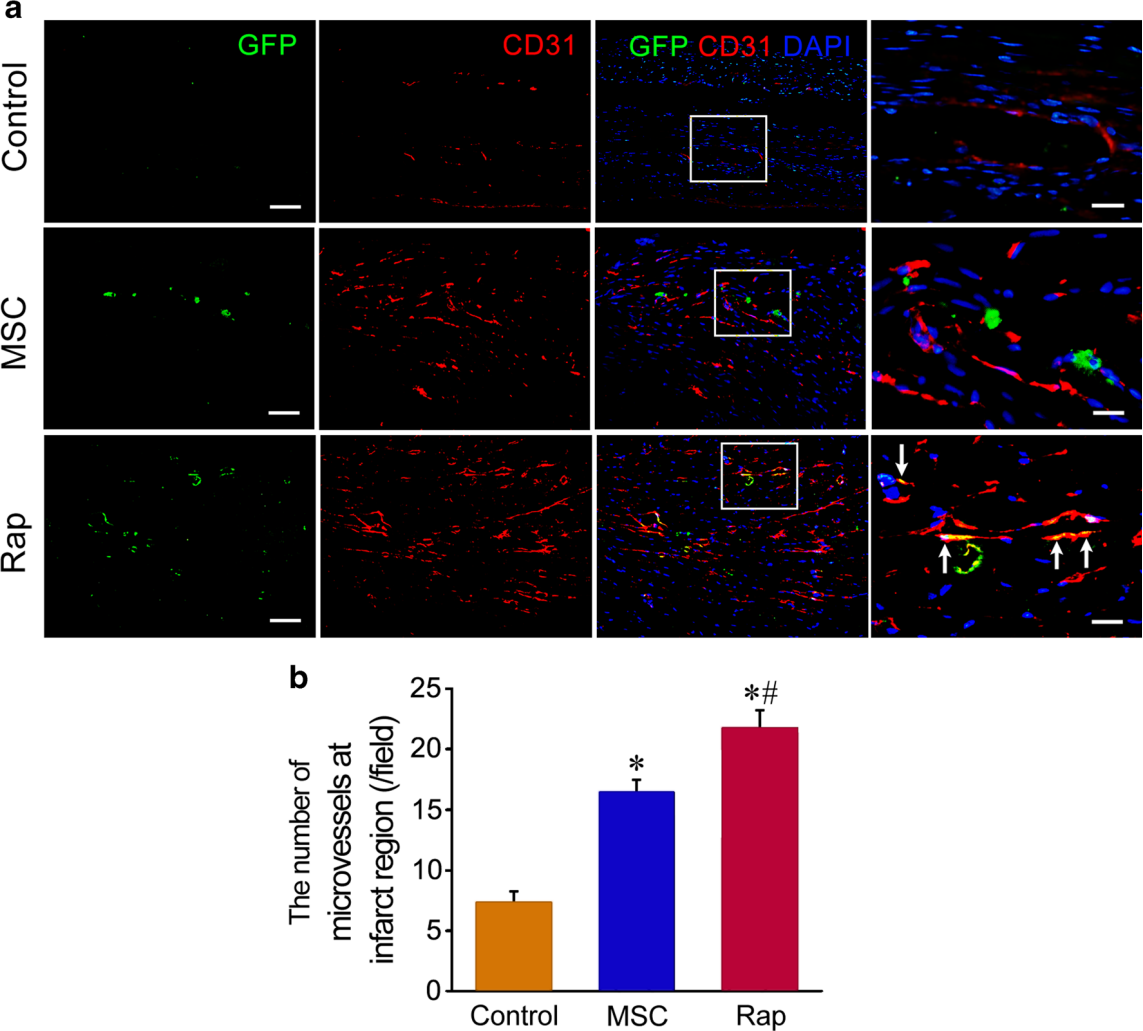
Regeneration of the microvessels after cell transplantation. **a** The microvessels at the infarct region. In rapamycin group, some of GFP^+^ cells express CD31, which are located on the wall of the microvessels (arrows). The panels of the fourth row are magnification of the boxes in the panels of the third row. Scale bar = 100 μm (the panels of the first to third rows) and 20 μm (the panels of the fourth row). **b** Statistic result of the number of the microvessels at the infarct region. *n* = 6. *p < 0.01 versus control group; ^#^*p <* 0.01 versus MSC group

At 4 weeks after transplantation, the density of the microvessels at the infarct region increased significantly in MSC and rapamycin groups. The number of the microvessels in rapamycin group was greater than that in MSC group. GFP and CD31 double immunostaining showed that some GFP^+^ cells expressed CD31 in rapamycin group. GFP^+^CD31^+^ cells were located on the wall of the microvessels (Fig. 6).

## Discussion

In this study, we demonstrate that rapamycin can activate autophagy of MSCs effectively. After treatment with rapamycin, LC3-II expression was upregulated, and LC3-positive puncta and autophagic ultrastructures was increased. LC3 includes two forms, LC3-I is cytosolic, while LC3-II is expressed specifically on the membrane of the autophagic structures. Therefore, LC3-II expression provides an accurate measure of autophagic flux [26]. Moreover, lysosomes in rapamycin-treated cells also increased significantly. Effective biogenesis of lysosomes is implicated in meeting the demand of autophagy activated with rapamycin. Rapamycin was originally isolated from the soil bacterium *Streptomyces hygroscopicus* in 1975, and possesses potent antifungal and immunosuppressive properties [27]. Mammalian target of rapamycin (mTOR), a serine/threonine kinase, is a master regulator of cellular metabolism and growth. Rapamycin forms a complex with FKB12, and this complex specifically binds to mTOR complex 1 (mTORC1) [19]. mTORC1 is acutely sensitive to inhibition by rapamycin (with a half maximal inhibitory concentration (IC50) in the nanomolar range) [27]. Lower dose (50% dose producing immunosuppressive drug level) of rapamycin can attenuate progressive ventricular dysfunction and remodeling in heart failure [28]. In this study, autophagy activated with low concentration of rapamycin is mild and safe for survival of MSCs.

The results of this study show that transient pretreatment with rapamycin promotes survival and differentiation the engrafted MSCs in I/R myocardium. After engraftment of rapamycin-pretreated MSCs, repair of the infarcted myocardium and restoration of cardial function were increased dramatically. In short-term or long-term of hypoxia and serum deprivation, rapamycin-activated autophagy protected the cells against apoptosis. Tracing of GFP and *Sry* gene shows that the survival of rapamycin-pretreated cells in the ischemic myocardium is enhanced significantly. Moreover, the results of 3-MA inhibition indicate that basic autophagy of the cells also has cytoprotective effect in the conditions of hypoxia and serum deprivation or in the ischemic myocardium. In addition, some cells in rapamycin-pretreated cells differentiated into cardiomyocytes or endothelial cells, which contributed to cardiomyogenesis and angiogenesis. Survival and differentiation of the engrafted cells within hostile ischemic and inflammatory microenvironment are limited [29]. Pre-activation of autophagy may extend application of MSC therapy in repair of the infarcted myocardium. Although it is impossible that rapamycin induces MSC differentiation towards cardiomyocytes, pretreatment with rapamycin may maintain higher cell viability and enable myocardial differentiation under induction of cytokines such as bone morphogenetic protein-2 [30]. Recent studies have suggested that autophagy can protects MSCs from apoptosis under hypoxia/serum deprivation [31] and against tumor necrosis factor-α-induced apoptosis [32]. Autophagy removes the damaged mitochondria, thereby reducing ROS production and DNA damage and inhibiting the release of proapopototic factors such as cytochrome c [33]. MSCs exhibit a high level of constitutive autophagy, while this basal autophagy decreases during differentiation of the cells [34]. Rapamycin promotes adhesion and differentiation of induced pluripotent stem cells [35]. Additionally, rapamycin enhances cell migration and optimize immunosuppressive potential of MSCs [36]. Therefore, we suggest that autophagy preactivated with rapamycin may serve as adaptive and resistant process of the engrafted MSCs against ischemic and inflammatory microenvironment in the infarcted myocardium.

A recent study has suggested that rapamycin protects myocardium against I/R injury [37]. Precondition with rapamycin is beneficial to protect the isolated heart and cardiomyocytes against I/R injury [38]. Acute ischemia stimulates autophagy which is primarily responsible for maintaining energy production, while the prolonged ischemia causes inhibition of autophagic flux. Restoration of oxygen on reperfusion leads to a significant increase in ROS production, which may impair autophagy flux [39, 40]. It is unknown whether local injection or sustained release from biomaterials in rapamycin administration is reliable for priming stem cells. Because magnitude of autophagy activation can not readily be assessed in vitro, no autophagy-specific surrogate endpoint currently exists, and optimal dosage and duration of treatment are also unknown [41, 42]. In our experiment, MSCs were primed with rapamycin before transplantation and then implanted at border of the infarct region, which were benefit for rapamycin-pretreated cells to survive avoid excess autophagy.

Our experimental data show that paracrine of rapamycin-pretreated MSCs and the myocardium may account for myocardial regeneration and angiogenesis after transplantation. In recent years, attention has been paid on paracrine mechanisms of reparative and regenerative effects of stem cells [43]. The medium conditioned by hypoxic MSCs reduces cardiomyocyte apoptosis and necrosis [44]. MSCs can release antiapoptotic or prosurvival factors during serum deprivation [45]. In our experiment, release of prosurvival or growth factors from rapamycin-pretreated cells was increased in the condition of hypoxia and serum deprivation. After transplantation of rapamycin-pretreated cells, production of prosurvival or growth factors was enhanced, while production of proinflammatory cytokines is suppressed in the myocardium. The factors released from the cells may play important roles in protecting I/R-injured myocardium, reducing inflammatory response, stimulating angiogenesis and recruiting endogenous stem cells. Only mRNA levels in paracrine of rapamycin-treated cells were examined in this study, while the protein levels of the cytokines released the cells need to be determined in further study. MSCs also secrete exosomes and microvesicles, promoting repair of the myocardium [46, 47]. Secretion of exosomes and microvesicles from autophagy-activated MSCs is deserved to be investigated.

## Conclusions

This study suggests that transplantation of rapamycin-pretreated stem cells can promote repair of the infarcted myocardium and improvement of cardiac function effectively. Rapamycin-preactivated autophagy enhances survival and differentiation of the transplanted stem cells in the hostile MI microenvironment. Paracrine of the engrafted cells and the injured myocardium may be involved in cardio-myogenesis and angiogenesis. Rapamycin has been approved by US Food and Drug Administration for use in organ transplantation. Rapamycin administration before stem cell transplantation is safe. Degree of autophagy activation can be controlled by adjusting concentration of rapamycin and duration of treatment. Therefore, pretreatment with rapamycin is an optimal strategy for stem cell therapy.

## Electronic supplementary material

ESM 1 (DOC 2267 kb)
