# Supplementary material for: Rapamycin-Preactivated Autophagy Enhances Survival and Differentiation of Mesenchymal Stem Cells After Transplantation into Infarcted Myocardium
Source: Stem Cell Rev Rep. 2020 Jan 11;16(2):344–56. doi: 10.1007/s12015-020-09952-1 (PMC7152587; doi:10.1007/s12015-020-09952-1)
Supplement: Supplementary file 1 — (DOC 2267 kb) [file 12015_2020_9952_MOESM1_ESM.doc]

**SUPPLEMENTAL MATERIAL**

**Rapamycin-preactivated autophagy enhances survival and differentiation of mesenchymal stem cells after transplantation into infarcted myocardium**

Zhi-hua Li, Yong-li Wang, Hai-jie Wang*, Jin-hong Wu, Yu-zhen Tan*

Department of Anatomy, Histology and Embryology, Shanghai Medical School of Fudan University, Shanghai 200032, China

**Supplementary Figure and Figure Legend**

**
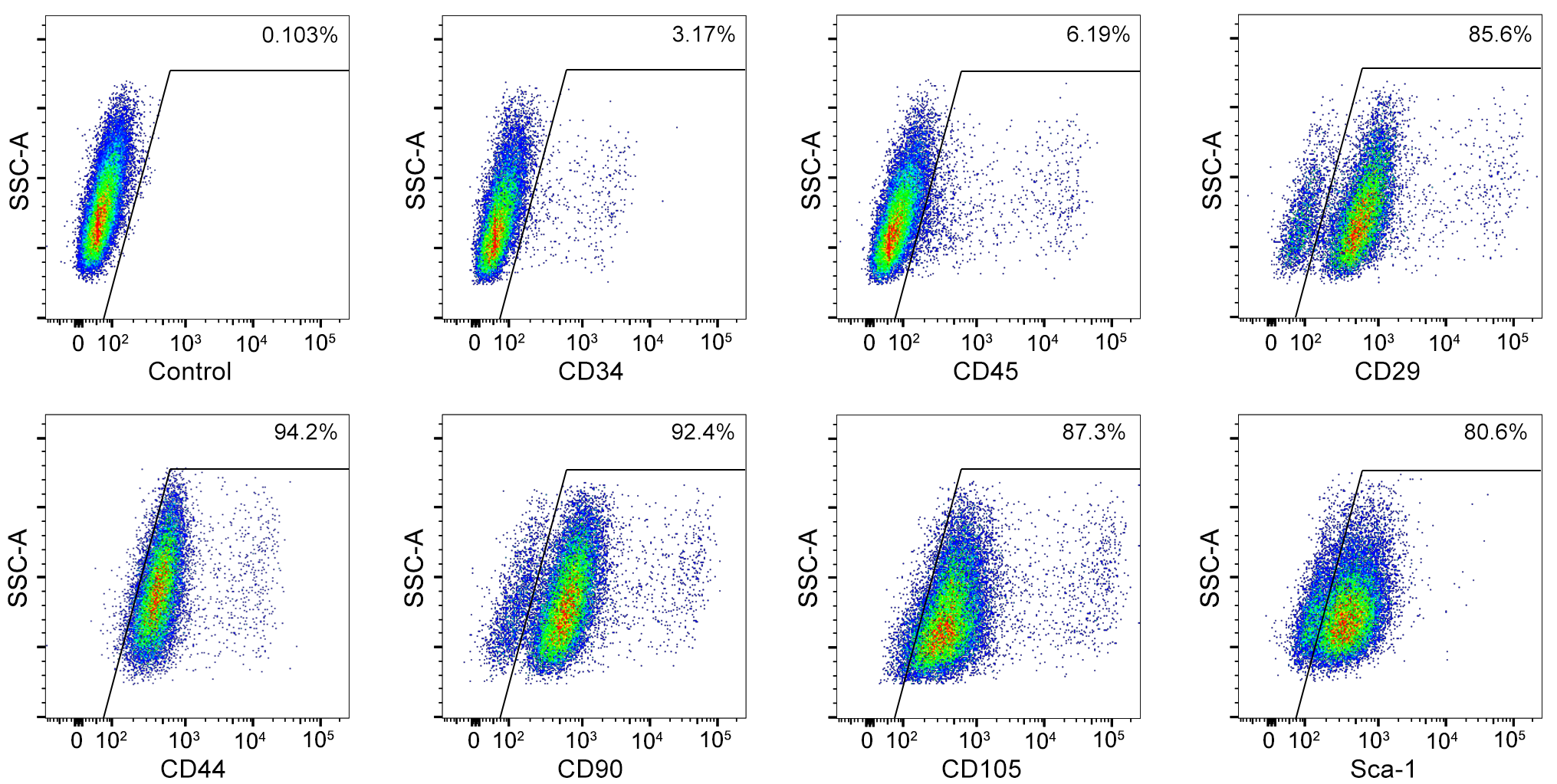
**

**Figure. S1** Phenotypic characteristics of MSCs isolated from rat bone marrow. The cells express CD29, CD44, CD90, CD105 and Scal-1, and are negative for expression of CD34 and CD45. Flow cytometric analysis.

**
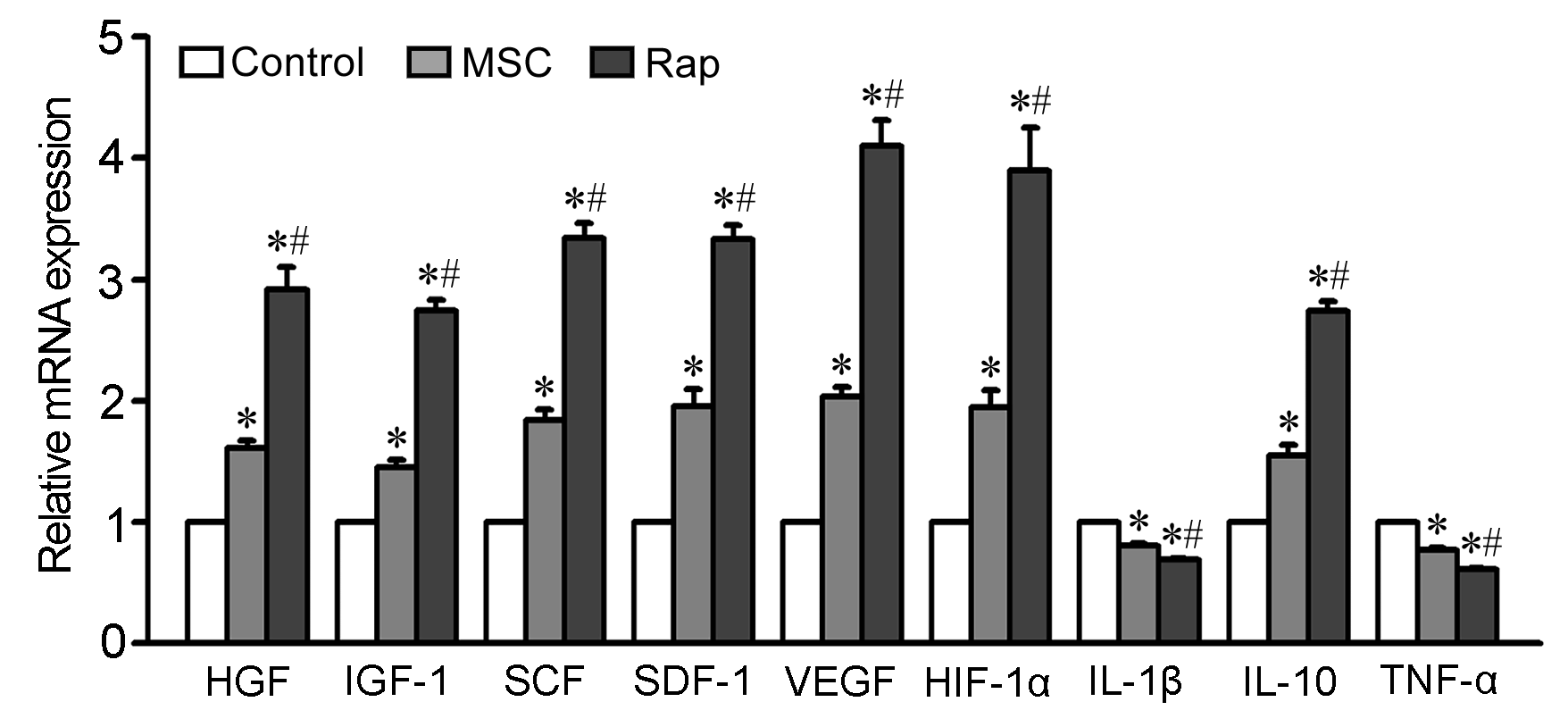
**

**Figure S2. Expression of paracrine factor mRNA in the infarcted myocardium after transplantation.** At seven days after transplantation, expression of *HGF*, *IGF-1*, *SCF*, *SDF-1*, *VEGF*, *HIF-1α, IL-1β, IL-10* and *TNF-α* in the infarcted myocardium was analyzed by qRT-PCR. All expression levels were normalized to that in the control group, which was assigned a value of 1. Data are represented as mean ± SD. **p* < 0.01 versus control group; #*p <* 0.01 versus MSC group. n = 3.


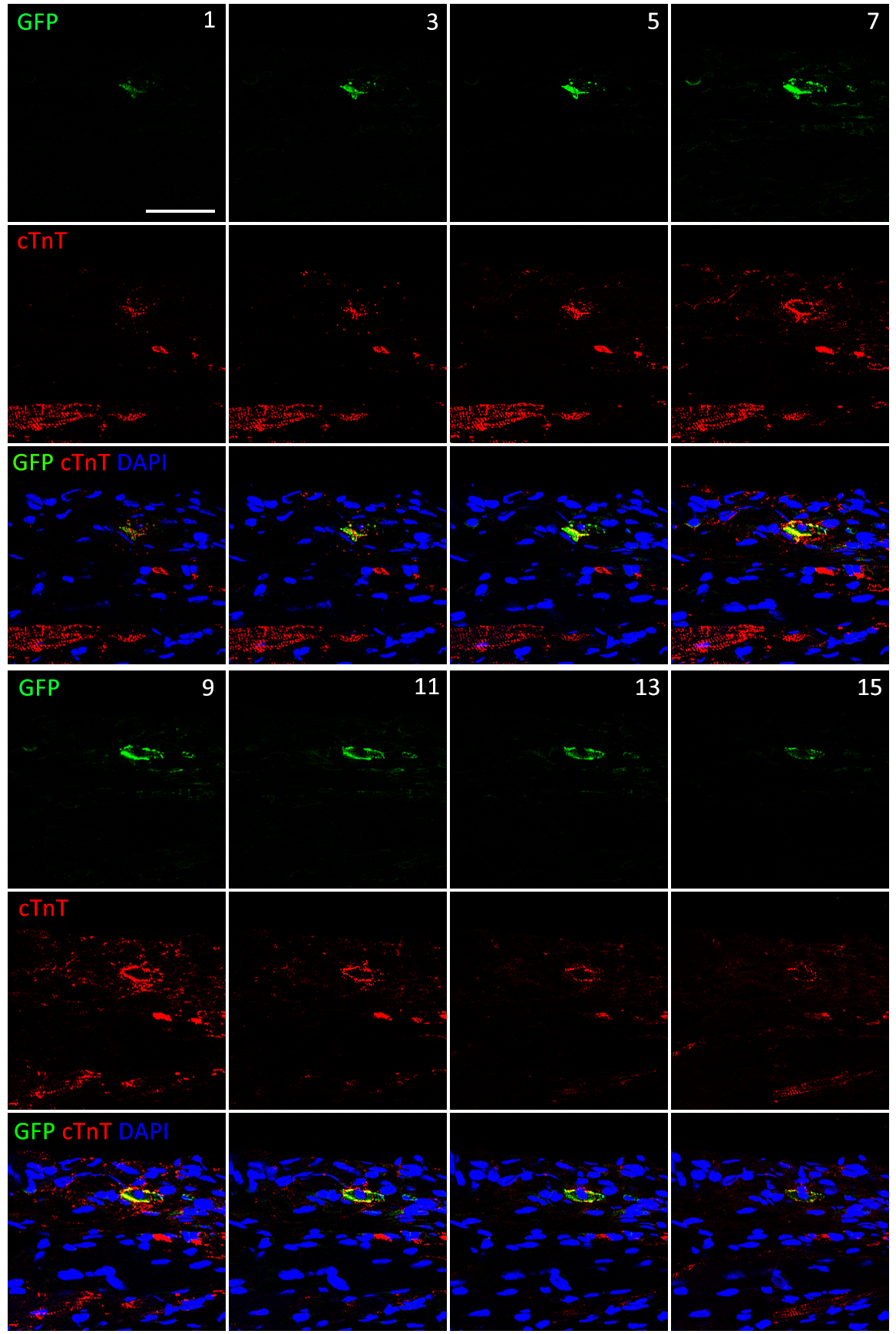


**Fig. S3** The cardiomyocyte differentiated from rapamycin-pretreated cells at four weeks after transplantation. The cardiomyocyte co-expressing GFP (green) and cTnT (red) was scanned with a confocal laser scanning microscope. Scale bar = 50 µm.

**Supplementary Table**

**Table S1 The Sequences of the primers.**

Table. Sequences of primers

| **Symbol** | | **Version** | **Sequences (5’ to 3’)** | **Length** |
| --- | --- | --- | --- | --- |
| HGF | NM_017017.2 | | (F) CCCGTTGTGAAGGAGATA  (R) CAAACTAACCATCCACCC | 134 bp |
| HIF-1α | NM_024359.1 | | (F) AAGTCTAGGGATGCAGCA  (R)CAAGATCACCAGCACCTAG | 175 bp |
| IGF-1 | NM_001082477.2 | | (F) CGCACCTCCAATAAAGAT  (R) AACTGAAGAGCGTCCACC | 138 bp |
| IL-1β | NM_031512.2 | | (F)TTCTTTGAGGCTGACAGACC  (R)CGTCTTTCATCACACAGGAC | 123 bp |
| IL-10 | NM_012854.2 | | (F)TGCCTTCAGCAGGAGTGAAG  (R)GGGAAGAAATCGATGACAG | 256 bp |
| SCF | NM_021843.4 | | (F) GGATGACCTCGTGGCATGTA  (R) GCCACCATGAAGTCCTTGAA | 147 bp |
| SDF-1 | NM_022177.3 | | (F) GATTCTTTGAGAGCCATGTCGC  (R) CACACCTCTCACATCTTGAGCC | 193 bp |
| TNF-α | NM_012675.3 | | (F)CCCAGACCCTCACACTCAGAT  (R)TTGTCCCTTGAAGAGAACCTG | 215 bp |
| VEGF | NM_001287114.1 | | (F) CGAGACGCAGCGACAAGGCA  (R) ACCTCTCCAAACCGTTGGCACG | 171 bp |
| β-actin | NM_031144.3 | | (F) TGACCCAGATCATGTTTGAGA  (R) CAAGGTCCAGACGCAGGAT | 186 bp |
